# Born Too Soon: Care for the preterm baby

**DOI:** 10.1186/1742-4755-10-S1-S5

**Published:** 2013-11-15

**Authors:** Joy E Lawn, Ruth Davidge, Vinod K Paul, Severin von Xylander, Joseph de Graft Johnson, Anthony Costello, Mary V Kinney, Joel Segre, Liz Molyneux

**Affiliations:** 1 MARCH, London School Hygiene & Tropical Medicine, UK; 2 Saving Newborn Lives, Save the Children, Cape Town, South Africa; 3 Kwa-Zulu Natal Dept. of Health, Pietermartizburg, South Africa; 4 NNASA-Neonatal Nurses Association of Southern Africa, Durban, South Africa; 5 Congress of International Neonatal Nurses (COINN; 6 All India Institute for Medical Sciences, New Delhi, India; 7 World Health Organization, Geneva, Switzerland; 8 Save the Children and MCHIP, Washington DC, USA; 9 University College London, UK; 10 Saving Newborn Lives, Save the Children, Cape Town, South Africa; 11 Consultant to Bill & Melinda Gates Foundation, Seattle, WA, USA; 12 Queen Elizabeth Hospital, College of Medicine, Blantyre, Malawi

## Abstract

As part of a supplement entitled "Born Too Soon", this paper focuses on care of the preterm newborn. An estimated 15 million babies are born preterm, and the survival gap between those born in high and low income countries is widening, with one million deaths a year due to direct complications of preterm birth, and around one million more where preterm birth is a risk factor, especially amongst those who are also growth restricted. Most premature babies (>80%) are between 32 and 37 weeks of gestation, and many die needlessly for lack of simple care. We outline a series of packages of care that build on essential care for every newborn comprising support for immediate and exclusive breastfeeding, thermal care, and hygienic cord and skin care. For babies who do not breathe at birth, rapid neonatal resuscitation is crucial. Extra care for small babies, including Kangaroo Mother Care, and feeding support, can halve mortality in babies weighing <2000 g. Case management of newborns with signs of infection, safe oxygen management and supportive care for those with respiratory complications, and care for those with significant jaundice are all critical, and are especially dependent on competent nursing care. Neonatal intensive care units in high income settings are de-intensifying care, for example increasing use of continuous positive airway pressure (CPAP) and this makes comprehensive preterm care more transferable. For health systems in low and middle income settings with increasing facility births, district hospitals are the key frontier for improving obstetric and neonatal care, and some large scale programmes now include specific newborn care strategies. However there are still around 50 million births outside facilities, hence home visits for mothers and newborns, as well as women's groups are crucial for reaching these families, often the poorest. A fundamental challenge is improving programmatic tracking data for coverage and quality, and measuring disability-free survival. The power of parent's voices has been important in high-income countries in bringing attention to preterm newborns, but is still missing from the most affected countries.

**Declaration:**

This article is part of a supplement jointly funded by Save the Children's Saving Newborn Lives programme through a grant from The Bill & Melinda Gates Foundation and March of Dimes Foundation and published in collaboration with the Partnership for Maternal, Newborn and Child Health and the World Health Organization (WHO). The original article was published in PDF format in the WHO Report "Born Too Soon: the global action report on preterm birth" (ISBN 978 92 4 150343 30), which involved collaboration from more than 50 organizations. The article has been reformatted for journal publication and has undergone peer review according to *Reproductive Health*'s standard process for supplements and may feature some variations in content when compared to the original report. This co-publication makes the article available to the community in a full-text format.

## Preterm baby survival and care round the world

Each year 15 million babies are born preterm and their survival chances vary dramatically around the world [1]. For the 1.2 million babies born in high income countries, increasing complexity of neonatal intensive care over the last quarter of the 20th century has changed the chances of survival at lower gestational ages. Middle-income and emerging economies have around 3.8 million preterm babies each year, and whilst some countries such as Turkey and Sri Lanka have halved deaths for preterm babies within a decade, other countries have made minimal progress [2]. South Asia and sub-Saharan Africa account for almost two-thirds of the world's preterm babies and over three-quarters of the world's newborn deaths due to preterm birth complications [1]. Worldwide, almost half of preterm babies are born at home, and even for those born in facilities, essential newborn care is often lacking.

This paper is the fifth in a supplement entitled "Born Too Soon". Previous papers in this series have outlined the policy context [3], epidemiology [4], and interventions preconceptually [5] and during pregnancy [6]. In this paper we focus on care of preterm newborns.

**Figure 1:**
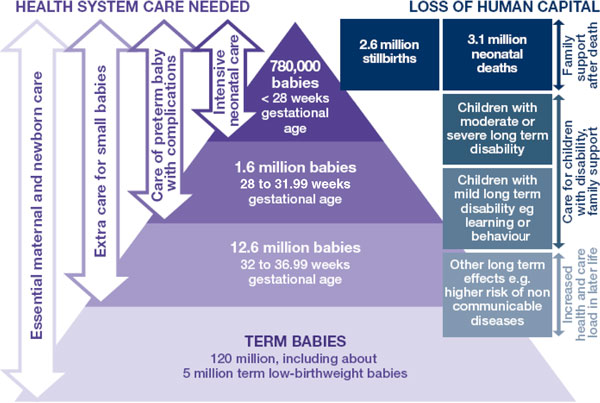
**135 million newborns and 15 million premature babies-health system needs and human capital outcomes around the year 2010**. Source: Born Too Soon report, chapter 5 [113]. Analysis using data from Blencowe et al., 2012 [1]; Cousens et al., 2011 [114]; Liu et al., 2012 [8].

We apply the simple WHO definition of all babies born at less than 37 weeks gestation, noting that this includes both provider initiated and spontaneous preterm birth, and many varying causations [4]. Most premature babies (>80%) are born between 32 and 37 weeks of gestation (moderate/late preterm), and many die needlessly for lack of simple, essential care such as warmth and feeding support (Figure 1). About 10% of preterm babies are born 28 to <32 weeks gestation, and in low-income countries more than half of those will die but many could be saved with feasible care, not including intensive care such as ventilation (Figure 1). For babies born before 28 weeks gestation, intensive care would be needed to save most of these, but it is important to realise that these are the minority - about 5% of premature babies. Yet in many countries, families and health care providers still perceive the deaths of any premature baby as inevitable.

In contrast, in high-income settings neonatal survival is extending to lower and lower extremes of gestational age. In 1990, few babies under 25 weeks gestation were surviving; yet by 2010, 95% of preterm babies under 28 weeks survived, and more than half of the babies born before 25 weeks gestation survived, although the latter have a higher risk of impairment [7].

**Figure 2:**
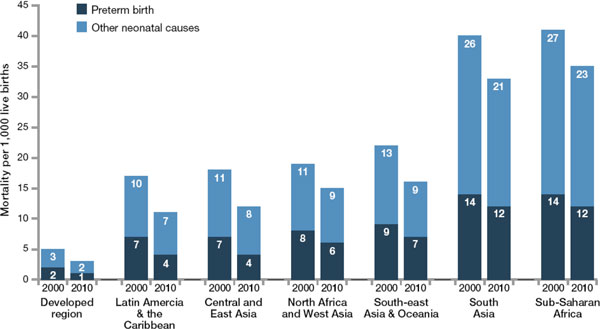
**Increasing survival gap for preterm babies around the world: Regional variation in preterm birth as direct cause of neonatal deaths showing change between 2000 to 2010**. Source: Born Too Soon, Chapter 5 [113]. Data from Child Health Epidemiology Reference Group and World Health Organization estimates of neonatal causes of death (Liu et al. 2012) [8].

Over the last few decades the survival gap for babies born in high-income countries and babies born in the poorest countries has widened dramatically, even though the pace of survival gains in high-income countries has slowed reaching the extremes of preterm gestation. For example, North America is still achieving an average annual reduction of more than 5% per year for preterm-specific mortality, yet Africa on average is improving mortality rates for preterm babies by only 1% a year (Figure 2). Those countries with the highest risk of death and the most feasible deaths to avert are still experiencing the least progress. The history of neonatal care in high-income countries shows that the major reduction in deaths occurred before neonatal intensive care was established. Yet the risk of a neonatal death due to complications of preterm birth is about twelve times higher for an African baby than for a European baby [8] (Figure 2).

An important but under-recognised issue for all countries is that of disability for survivors of preterm birth [1,9]. In the early days of neonatal intensive care, disabilities were common amongst survivors, ranging from some school learning disability through to severe cerebral palsy. Impairment outcomes have a heavy toll on families and on the health system. Indeed a recent report estimated that the average baby born 28 to 31 weeks gestation in the United States costs $95,000 in medical care in the first year alone [10]. Overtime the pattern of impairment from preterm birth in high-income countries has shifted. The focus of intensive care has shifted to extremely premature babies (less than 28 weeks), or "micro preemies", as this smaller subset of babies has increased risk and severity of impairment [7,11]. With the scale up of neonatal intensive care, a focus on follow up and family support is critical.

Recent data show that even late and moderate preterm (LAMP, or 32 to <37 weeks gestation) is also associated with significant adverse effects, including those on school learning, prompting increasing debate regarding avoidable causes of moderate preterm birth such as high caesarean birth rates [12,13]. These long-term effects on society and on the health system as well as more evidence of the link with non-communicable diseases in later adult life [3] underline that the importance of addressing preterm birth is beyond survival alone.

Over the last four decades with an increasing focus on evidence-based care for premature babies in high-income countries, the risk of long-term impairments is reducing. Neonatal intensive care has also become less interventionist and hence some aspects are also potentially more feasible to adapt to lower-income settings. There have been notable advances in quality of intensive care for premature babies.

Widespread use of antenatal corticosteroids in high-and some middle-income countries for mothers at gestation of 32 weeks or less, following multiple RCTs and the National Institute of Health consensus statement [14], ensuring that babies are less likely to develop respiratory distress syndrome (RDS), or have less severe RDS [15-17]. All trials have been conducted in settings where intensive or special care for preterm infants is available. While the effect has biological plausibility, the magnitude of effect in low-income countries without intensive care is uncertain, although a meta-anlsysis for middle-income country trials showed a greater effect than in high-income settings. An NIH sponsored trial in low- and middle-income settings is ongoing.

A shift to less intensive ventilator pressures and increasing use of continuous positive airway pressure (CPAP), now often the respiratory support method of choice [18].

Detailed quality of care protocols and "job aids" for almost every aspect of care have improved quality and also shifted more care to the responsibility of skilled neonatal nurses, particularly with respect to addressing infection prevention, feeding support, use of intravenous fluids, and safe oxygen use with careful tracking of oxygen saturation levels and follow-up services [19].

Deliberate attention to baby friendly care, reducing pain and over stimulation and more family friendly care, including family rooms linked to neonatal units and increased access for parents to their babies while in neonatal care units [20].

In low- and middle-income countries, there are limited comparable data on long-term outcomes after preterm birth [21,22]. However, small studies suggest a high risk of moderate or severe neurodevelopmental impairment and an urgent need to improve awareness, data and care. Retinopathy of prematurity caused an epidemic of blindness for preterm babies in Europe and North America 50 years ago, especially after high or unmonitored use of oxygen. Data from Latin America show increasing rates of retinopathy of prematurity [23,24] and it is likely that areas without data such as Southeast Asia are also experiencing an increase, recreating an avoidable problem. As neonatal care is improved and complexity increases, monitoring quality of care and tracking impairment outcomes are critical and should not be considered an optional extra in low-resource settings. Urgent attention is needed to develop standard, simpler measures of such impairments, to integrate these metrics into other measurement systems, and to provide support for such babies and their families [21].

## Priority packages and evidence-based interventions

**Figure 3:**
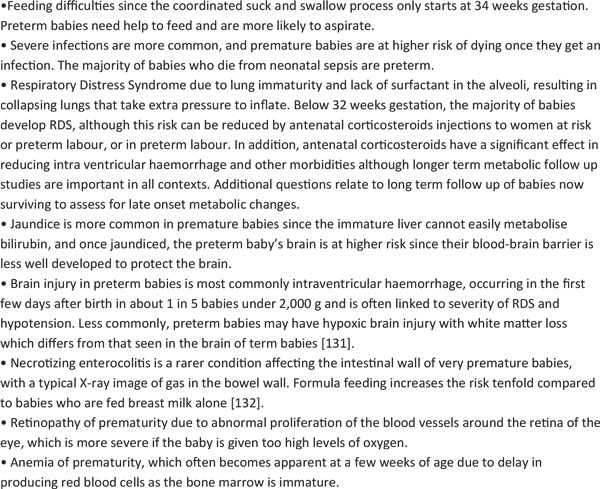
**Preterm babies face specific risks**. Source: Born Too Soon, Chapter 5 [113].

All newborns are vulnerable given that birth and the following few days hold the highest concentrated risk of death of any time in the human lifespan. Every baby needs essential newborn care, ideally with their mothers providing warmth, breastfeeding and a clean environment. Premature babies are especially vulnerable to temperature instability, feeding difficulties, low blood sugar, infections, and breathing difficulties (Table 1). There are also complications that specifically affect premature babies (Figure 3).

**Table 1:** Life-saving essential and extra newborn care.

Risk for all babies, especially those who are preterm	Essential care for all babies	Extra care for preterm babies
*Hypothermia = low body temperature *(increased risk of infections, mortality and for preterm babies increased risk of RDS)	*Thermal care* Drying, warming, skin-to-skin and delayed bathing	*Extra thermal care* Kangaroo Mother Care, baby hats, blankets, overhead heaters, incubators
*Cord and skin infections, neonatal sepsis*	*Hygienic cord and skin care at birth and home care practices* Hand washing and other hygiene Delayed cord clamping Consider chlorohexifine	*Extra attention to infection prevention and skin care* Consider chlorohexidine and emolients
*Hypoglycemia = low blood sugar* (Increased risk of impairment or death)	*Early and exclusive breastfeeding*	*Extra support for breastfeeding* e.g. expressing and cup or tube feeding, supplemented breast milk if indication Lack of breast milk is a risk factor for necrotizing entereocolitis in preterm babies
*Hypoxia = low oxygen levels* (Increased risk of impairment or death for preterm babies, higher risk of RDS and intracranial bleeding)	*Neonatal resuscitation if not breathing at birth* Bag-and-mask resuscitation with room air is sufficient for >99% of babies not breathing at birth	*Safe oxygen use* Monitored oxygen use e.g. in head box or with nasal cannula, routine use of pulse oximeters

Source: : Born Too Soon, Chapter 5 [113].

Saving lives and preventing disability from preterm birth can be achieved with a range of evidence-based care increasing in complexity and ranging from simple care such as warmth and breastfeeding up to full intensive care (Table 2). The packaged interventions in this chapter are adapted from a recent extensive evidence review and a consensus report, "Essential Interventions Commodities and Guidelines for Reproductive Maternal, Newborn and Child Health" [25].

**Table 2:** Priority evidence-based packages and interventions for preterm babies.

	Grade
**Essential Newborn Care For All Babies**	
Thermal care (drying, warming, skin-to-skin and delayed bathing) Hygienic cord and skin care Early initiation, exclusive breastfeeding	Evidence: Low to moderate Recommendation: Strong
Neonatal resuscitation for babies who do not breathe at birth	Evidence: Low to moderate Recommendation: Strong
**Extra Care For Small Babies**	
Kangaroo Mother Care for small babies (birthweight <2,000 g) Extra support for feeding	Evidence: Moderate to high Recommendation: Strong
**Care For Preterm Babies With Complications**	
Case management of babies with signs of infection Safe oxygen management and supportive care for RDS Case management of babies with significant jaundice	Evidence: Moderate to high* Recommendation: Strong
Hospital care of preterm babies with RDS including if appropriate, CPAP and/or surfactant	Evidence: Moderate to high* Recommendation: Strong
Intensive neonatal care	Evidence: High* Recommendation: Strong

Source: Born Too Soon, Chapter 5 [113] adapted from The Healthy newborn: A reference guide for program managers (Lawn et al., 2001) [39], and PMNCH essential Interventions (PMNCH, 2011) [127] using WHO guidelines, LiST, Cochrane and other reviews, with detailed references in text. *Note that the evidence is mostly from high-income countries and more context specific research required in middleand low-income settings.

Recognition of small babies and distinguishing which ones are preterm are essential first steps in prioritising care for the highest risk babies. First trimester ultrasound assessment is the most accurate measure, but this is not available for most of the world's pregnant women [4]. Other options include Last Menstrual Period, using birthweight as a surrogate or assessment of the baby to estimate gestational age (e.g., Dubowitz or other simpler scoring methods). The highest-risk babies are those that are both preterm and growth restricted.

## Package 1: Essential and extra newborn care

Care at birth from a skilled provider is crucial for both women and babies and all providers should have the competencies to care for both mother and baby, ensuring that mother and baby are not separated unnecessarily, promoting warmth, early and exclusive breastfeeding, cleanliness and resuscitation if required [26]. These practices are essential for full-term babies, but for premature babies, missing or delaying any of this care can rapidly lead to deterioration and death. For all babies at birth, minutes count.

### Thermal care

Simple methods to maintain a baby's temperature after birth include drying and wrapping, increased environmental temperature, covering the baby's head (e.g., with a knitted cap), skin-to-skin contact with the mother and covering both with a blanket [27,28]. Delaying the first bath is promoted, but there is a lack of evidence as to how long to delay, especially if the bath can be warm and in a warm room [29]. Kangaroo Mother Care (KMC) has proven effect on mortality for babies <2,000 g and is discussed below. Equipment-dependent warming techniques include warming pads or warm cots, and radiant heaters or incubators; however, these require additional nursing skills and careful monitoring [28]. Sleeping bags lack evidence for comparison with skin-to-skin care or of large-scale implementation. There are several trials suggesting benefit for plastic wrappings but, to date, these have been tested only for extremely premature babies in neonatal intensive care units [30].

### Feeding support

At the start of the 20th century, Pierre Budin, a French obstetrician, led the world in focusing on the care of "weaklings," as premature babies were known then. He promoted simple care - warmth, breastfeeding and cleanliness. However, by the middle of the 20th century, formula milk was widely used and the standard text books said that premature babies should not be fed for the first few days. After 1960, the resurgence of attention and support for feeding of premature babies was an important factor in reducing deaths before the advent of intensive care [31].

Early initiation of breastfeeding within one hour after birth has been shown to reduce neonatal mortality [32-34]. Premature babies benefit from breast milk nutritionally, immunologically and developmentally [35]. The short-term and long-term benefits compared with formula feeding are well established with lower incidence of infection and necrotising enterocolitis and improved neuro-developmental outcome [36,37]. Most premature babies require extra support for feeding with a cup, spoon or another device such as gastric tubes (either oral or nasal) [38,39]. In addition, the mother requires support for expressing milk. Where this is not possible, donor milk is recommended [38]. In populations with high HIV prevalence, feasible solutions for pasteurisation are critical. Milk-banking services are common in many countries and must be monitored for quality and infection prevention. Extremely preterm babies under about 1,000 g and babies who are very unwell may require intravenous fluids or even total parenteral nutrition, but this requires meticulous attention to volume and flow rates. Routine supplementation of human milk given to premature babies is not currently recommended by WHO. WHO does recommend supplementation with vitamin D, calcium and phosphorus and iron for very low birthweight babies [38] and vitamin K at birth for low birthweight babies [40,41].

### Infection prevention

Clean birth practices reduce maternal and neonatal mortality and morbidity from infection-related causes, including tetanus [42]. Premature babies have a higher risk of bacterial sepsis. Hand cleansing is especially critical in neonatal care units. However basic hygienic practices such as hand washing and maintaining a clean environment are well known but poorly done. Unnecessary separation from the mother or sharing of incubators should be avoided as these practices increase spread of infections. For the poorest families giving birth at home, the use of clean birth kits and improved practices have been shown to reduce mortality [43]. Cluster trials of participatory learning through women's groups have shown large reductions in both maternal and neonatal mortality, with increased handwashing by birth attendants and increased use of clean delivery kits [44].

Recent cluster-randomised trials have shown some benefit from chlorhexidine topical application to the baby's cord and no identified adverse effects. To date, about half of trials have shown a significant neonatal mortality effect especially for premature babies and particularly with early application, which may be challenging for home births [45-47]. Another possible benefit of chlorhexidine is a behaviour change agent -- in many cultures around the world, something is applied to the cord and a policy of chlorhexidine application may accelerate change by substituting a harmful substance for a helpful one.

The skin of premature babies is more vulnerable, and is not protected by vernix like a term baby's. Topical application of emollient ointment such as sunflower oil or Aquaphor™ reduces water loss, dermatitis and risk of sepsis [48] and has been shown to reduce mortality for preterm babies in hospital-based trials in Egypt and Bangladesh [49,50]. Three trials are now testing the effect of emollients in community settings in South Asia, but as yet there are none being conducted in Africa [51]. This is a potentially scaleable, simple approach to save lives even where most births are at home.

Another effective and low cost intervention is appropriate timing for clamping of the umbilical cord, waiting 2-3 minutes or until the cord stops pulsating, whilst keeping the baby below the level of the placenta. For preterm babies this reduces the risk of intracranial bleeding and need for blood transfusions as well as later anemia. Yet this intervention has received limited attention [52]. Possible tension between delayed cord clamping and active management of the 3rd stage of labour with controlled cord traction has been debated, but the Cochrane review and also recent-evidence statements by obstetric societies support delayed cord clamping for several minutes in all uncomplicated births [53].

### Package 2: Neonatal resuscitation

Between 5 to 10% of all newborns and a greater percentage of premature babies require assistance to begin breathing at birth [54]. Basic resuscitation through use of a bag-and-mask or mouth-to-mask (tube and mask) will save four out of every five babies who need resuscitation; more complex procedures, such as endotracheal intubation, are required only for a minority of babies who do not breathe at birth and who are also likely to need ongoing ventilation. Recent randomised control trials support the fact that in most cases assisted ventilation with room air is equivalent to using oxygen, and unnecessary oxygen has additional risks [55]. Expert opinion suggests that basic resuscitation for preterm births reduces mortality by about 10% in addition to immediate assessment and stimulation [56]. An education program entitled Helping Babies Breathe has been developed by the American Academy of Pediatrics and partners for promotion of basic neonatal resuscitation at lower levels of the health system in low-resource settings and is currently being scaled up in over 30 lowincome countries and promises potential improvements for premature babies [2,57-59]. Whether communitybased resuscitation training will reduce neonatal mortality is much less certain [60,61].

### Package 3: Kangaroo Mother Care

KMC was developed in the 1970s by a Colombian paediatrician, Edgar Rey, who sought a solution to incubator shortages, high infection rates and abandonment among preterm births in his hospital [62,63]. The premature baby is put in early, prolonged and continuous direct skin-to-skin contact with her mother or another family member to provide stable warmth and to encourage frequent and exclusive breastfeeding. A systematic review and metaanalysis of several randomised control trials found that KMC is associated with a 51% reduction in neonatal mortality for stable babies weighing <2,000 g if started in the first week, compared to incubator care [64]. These trials all considered facility-based KMC practice where feeding support was available. An updated Cochrane review also reported a 40% reduction in risk of post-discharge mortality, about a 60% reduction in neonatal infections and an almost 80% reduction in hypothermia. Other benefits included increased breastfeeding, weight gain, mother-baby bonding and developmental outcomes [65]. In addition to being more parent and baby friendly, KMC is more health-system friendly by reducing hospital stay and nursing load and therefore giving cost savings [66]. KMC was endorsed by the WHO in 2003 when it developed a program implementation guide [67]. Some studies and program protocols have a lower weight limit for KMC, e.g., not below 800 g, but in contexts where no intensive care is available, some babies under 800 g do survive with KMC and more research is required before setting a lower cut off. Despite the evidence of its cost effectiveness, KMC is underutilised although it is a rare example of a medical innovation moving from the Southern hemisphere, with recent rapid uptake in neonatal intensive care units in Europe [64].

### Package 4: Special care of premature babies and phased scale up of neonatal intensive care

Moderately-premature babies without complications can be cared for with their mothers on normal postnatal wards or at home, but babies under 32 weeks gestation are at greater risk of developing complications and will usually require hospital admission. Fewer babies are born under 28 weeks of gestation and most of these will require intensive care.

### Care of babies with signs of infection

Improved care involves early detection of such danger signs and rapid treatment of infection, while maintaining breastfeeding if possible [68,69]. Identification is complicated by the fact that ill premature babies may have a low temperature, rather than fever. First level management of danger signs in newborns has relatively recently been added to Integrated Management of Childhood Illness guidelines [68,70]. WHO recommends that all babies with danger signs be referred to a hospital. Where referral is not possible, then treatment at the primary care centre can be lifesaving.

### Care of babies with jaundice

Premature babies are at increased risk of jaundice as well as infection, and these may occur together compounding risks for death and disability [22]. Since severe jaundice often peaks around day 3, the baby may be at home by then. Implementation of a systematic predischarge check of women and their babies would be an opportunity to prevent complications or increase careseeking, advising mothers on common problems, basic home care and when to refer their baby to a professional.

### Babies with Respiratory Distress Syndrome

For premature babies with RDS, methods for administering oxygen include nasal prongs, or nasal catheters. Safe oxygen management is crucial and any baby on continuous oxygen therapy should be monitored with a pulse oximeter [71].

The basis of neonatal care of very premature babies since the 1990s was assisted ventilation. However, reducing severity of RDS due to greater use of antenatal corticosteroids and increasing concerns about lung damage prompted a shift to less intensive respiratory support, notably CPAP, commonly using nasal prongs to deliver pressurised, humidified, warmed gas (air and/or oxygen) to reduce lung and alveoli collapse [72]. This model of lower intensity may be feasible for wider use in middle-income countries and for some low-income countries that have referral settings with stronger systems of support such as high-staffing, 24-hour laboratories.

Recent trials have demonstrated that CPAP reduces the need for positive pressure ventilation of babies less than 28 weeks gestation, and the need for transfer of babies under 32 weeks gestation to neonatal intensive care units [73-75]. One very small trial in South Africa comparing CPAP with no ventilation among babies who were refused admission to neonatal intensive care units found CPAP reduced deaths [76]. In Malawi, a CPAP device developed for low-resource settings is being trialed in babies with respiratory distress who weigh over 1,000 g [77]. Early results show 67% of babies on CPAP survived compared to 24% without CPAP but on oxygen [78]. An important outcome will be to assess the nursing time required and costs [79].

Increasing use of CPAP without regulation is a concern. Many devices are in the "homemade" category; several low-cost bubble CPAP devices are being developed specifically for low-income countries but need to be tested for durability, reliability and safety [80]. CPAP-assisted ventilation requires adequate medical and nursing skill to apply and deliver safely and effectively, and also requires other supportive equipment such as an oxygen source, oxygen-monitoring device and suction machine.

Surfactant is administered to premature babies' lungs to replace the missing natural surfactant, which is one of the reasons babies develop RDS. The first trials in the 1980s demonstrated mortality reduction in comparison to ventilation alone, but it was 2008 before surfactant was added to the WHO Essential Medicine list [81]. Uptake is limited in middleand especially low-income countries as the current products can only be feasibly administered in a well-equipped and staffed hospital that can intubate babies. The cost also remains a significant barrier. In India, surfactant costs up to $600 for a dose [82]. Data from India and South Africa suggest that surfactant therapy is restricted to use in babies with potential for better survival, usually over 28 weeks gestation due to its high price [82]. Costs may be reduced by synthetic generics and simplified administration, for example with an aerosolised delivery system, but before wide uptake is recommended, studies should assess the additional lives saved by surfactant once antenatal corticosteroids and CPAP are used.

## Evidence limitations

Most published trials come from high-income countries where care for premature babies assumes the presence of neonatal intensive care, and large multi-site trials often examine the incremental effect of a specific change in care. Few rigorous trials are undertaken in lower-income settings where severe morbidity and fatal outcomes are common, contextual challenges may be critical and the counterfactual or control group should really be women or neonates receiving no care at all as this is the real question for policymakers. For example, in the KMC RCTs the control group was those receiving routine incubator care, which may dilute the impact measured compared to a counterfactual of no care. Ironically, more of the large recently funded rigorous trials are community-based, such as those assessing chlorhexidine and emollients [51], and there is an urgent need for more facility-based research addressing quality of care and including cost analyses.

There were a number of interventions considered in a systematic review of essential interventions that are used in high-income settings for premature babies but were not included in the global recommendations for scale up due to lack of context-specific evidence on cost effectiveness-for example, caffeine citrate to reduce the risk of apnea of prematurity [83]. Thus, more evidence from low-income settings is required particularly with respect to context-specific adaptation and associated implementation realities.

## Program opportunities for scale up of care

**Figure 4:**
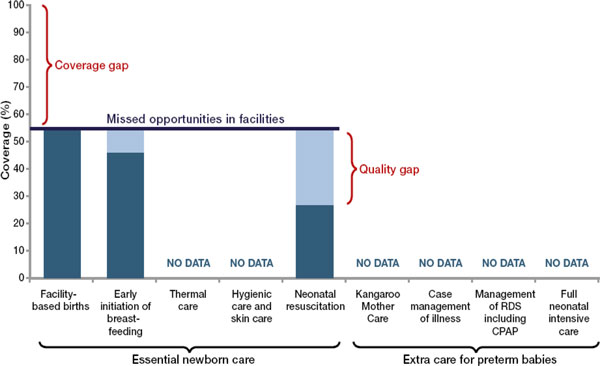
**Missed opportunities to reach preterm babies with essential interventions, median for Countdown to 2015 priority countries**. Source: Born Too Soon, Chapter 5 [113]. Data sources: Adapted (Kinney et al., 2010) [115] using data from UNICEF Global Databases (UNICEF, 2012) [116] based on Demographic Health Surveys, Multiple Indicator Cluster Surveys and other national surveys, neonatal resuscitation from LiST [117].

National coverage data for many of the evidence-based interventions for premature babies are lacking even in high-income settings, hence it is difficult to assess the global situation for care of premature babies or indeed for several important newborn care interventions (Figure 4).

For the 50 million home births without skilled care, the poorest women in the poorest countries, a major care gap is obvious. In sub-Saharan Africa, more than half of home births are alone, with no attendant [84]. In South Asia, around one-third of home births are without traditional birth attendants. In these instances, the primary caregivers of babies are their mothers and their families. Ensuring that women and communities are informed about healthy home care and enabled to care for their newborns and especially their preterm babies in the best possible way is critical. Women's groups which offer peer counselling and community mobilisation have been shown to have a significant effect on maternal and neonatal mortality [44,85,86].

The increasing pace of policy and program change for home-visit packages during pregnancy and after birth provides an opportunity to empower women to have a better outcome themselves and for their babies [87]. An early postnatal visit (within two days of birth) is one of only seven coverage indicators along the continuum of care selected by the United Nations Commission on Information and Accountability and tracked by Countdown to 2015 [88,89]. In the 75 Countdown to 2015 priority countries, only 1 in 3 women and babies have an early postnatal visit -- the lowest of the seven indicators. This early visit is critical for survival and health and an important opportunity to identify preterm babies. Novel methods for identification of premature babies include community health workers using foot size to identify those babies and then providing extra visits, breastfeeding support and referral to a facility if needed [90].

As well as gaps in coverage of crucial interventions for women and babies, there are equity gaps between rich and poor, public and private health sectors, provinces and districts and among rural, urban and peri-urban populations. Complex, facility-based interventions tend to have a higher level of inequity than simpler interventions that can be delivered closer to home [91]. For example, there is low inequity for immunisation and antenatal care, while higher disparities exist for skilled attendance coverage [92]. Among the 54 of 75 Countdown to 2015 priority countries with equity data, birth in a health facility is more than twice as likely for a richer family compared to a poorer family [93].

Many African and most South Asian countries are experiencing increases in health facility births, some very rapidly [89]. However, the quality of care has not kept pace with coverage, leaving a quality gap but also giving cost-effective opportunities for lifesaving care for women and babies who are reachable in health facilities. For example, midwives are skilled and equipped to provide essential newborn care and resuscitation if needed. However, often key commodities or attention to infection prevention are lacking. Perinatal audit data and process can be a powerful tool for improving quality of care and can also be collated and used for national or subnational improvement of care [94].

Figure 4 shows the coverage and quality gaps for premature baby care in the Countdown to 2015 priority countries, highlighting the data gaps. With just over 50% of all births taking place in health facilities, essential newborn care could be provided for all those babies. Yet data show even the apparently simple practices of hand cleansing and warmth in the labour room are poorly done around the world [95]. Early initiation of breastfeeding is tracked by national household surveys but the practices for premature babies and duration of breastfeeding for preterm babies is not known at national level.

Neonatal resuscitation scale up is benefitting from recent innovation in technology and from public-private partnerships [96] and also more attention since being listed as one of the 13 priorities for the United Nations Commission on Life Saving Commodities for Women and Children [97]. However, data from Service Provision Assessment surveys suggested that under half of all skilled birth attendants had resuscitation skills and/or the correct equipment in terms of bag-and-mask (Figure 4) [54].

**Figure 5:**
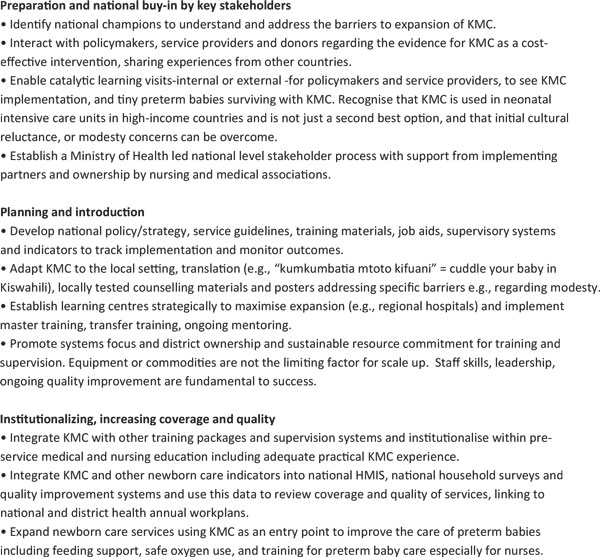
**Kangaroo Mother Care -what works to accelerate progress towards scale? Source: Born Too Soon, Chapter 5 **[113].

KMC, despite being established for more than 20 years, has had limited scale up (Figure 5). It is currently implemented on a large scale in only a few countries such as Colombia, Brazil and South Africa. There has also been rapid uptake in neonatal intensive care units in highincome countries, including for ventilated babies [98]. Systematic scale up of KMC is making progress in some countries in sub-Saharan Africa and South Asia including Malawi [99], Tanzania, Rwanda, Ghana [100], Indonesia and Vietnam [101]. In other countries, a KMC unit established in one teaching hospital over a decade ago has yet to benefit babies in the rest of the country. Lessons are being learned in overcoming barriers such as lack of knowledge by policy makers and service providers. Countries that are making more rapid progress have a national policy for KMC, a learning site, national champions, and a plan for national implementation. IN addition, they have integrated training along with essential newborn care and resuscitation into pre-service medical and nursing education (Figure 5). KMC can be safely delivered by trained patient attendants under the supervision of nurses, allowing nurses to look after the sickest neonates - a successful example of taskshifting [99]. A major impediment to program tracking and accountability is the lack of data for coverage of KMC, although this indicator could feasibly be tested for inclusion in household surveys.

**Figure 6:**
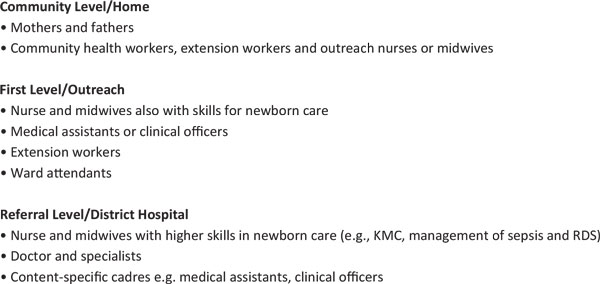
**The right people for reducing deaths and disability in preterm babies**. Source: Born Too Soon, Chapter 5 [113].

Quality of service provision requires the availability of people with the right skills (Figure 6) as well as essential equipment and drugs. Indeed, for newborn survival, skilled people are at least as critical as equipment and commodities (Table 3) [102]. Shortages of qualified health workers and inadequate training and skills for the care of premature babies are a major reason for poor progress in reducing neonatal deaths [92,103]. Nurses or midwives with skills in critical areas such as resuscitation, KMC, safe oxygen management, and breastfeeding support are the frontline worker for premature babies, yet in the whole of sub-Saharan Africa there are no known neonatal nurse training courses. Urgent systematic attention is required for pre-service and in-service training, non-rotation of nurses with skills in neonatal care, and where appropriate the development of a neonatal nurse cadre, as well as rewarding for those who work against the odds in hard-to-serve areas [2].

**Table 3:** Tools, technologies, and innovations required for the care of preterm babies.

Priority packages and interventions	Current technology/Tools	Technological innovations required
**All babies**		
Essential newborn care and extra care forpreterm babies • Thermal care (drying, warming, skin-to-skin and delayed bathing) • Early initiation, exclusive breastfeeding • Hygienic cord and skin care	• Protocols for care, training materials and job aids • Materials for counselling, health education and health promotion • Weighing scales • Cord clamp and scissors, clean birth kit if appropriate • Vitamin K for LBW babies	• Generic communications and counselling toolkit for local adaptation • Generic, modular training kit for adaptation, novel methods e.g. cell phone prompts • Birth kits for frontline workers • Chlorhexidine preparations for application to the umbilical cord • Simplified approaches to identifying preterm babies such as footsize
Neonatal resuscitation for babies who do not breathe at birth	• Materials for training and job aids • Training manikins • Newborn resuscitation devices (bag-and-mask) • Suction devices • Resuscitation stations with overhead heater • Clock with large face and second hand	• Wide scale novel logistics systems to increase availability of devices for basic resuscitation and training manikins • Additional innovation for resuscitation devices (e.g. upright bag-and-mask, adaptable, lower cost resuscitation stations)
**Preterm babies**		
Kangaroo mother care for small babies(birthweight <2,000 g)	• Cloth or wrap for KMC • Baby Hats	Generic communications and counselling toolkit for local adaptation, Innovation to address cultural, professional barriers Generic, modular training kit and job aids for local adaptation
Care of preterm babies with complications including: • Extra support for feeding preterm and small babies • Case management of babies with signs of infection • Safe oxygen management and supportive care for RDS • Case management of babies with significant jaundice • Managing seizures	• Nasogastric tubes, feeding cups, breast milkpumps • Blood sugar testing sticks • IV fluids including glucose and more accurate giving sets • Syringe drivers • Injection antibiotics, 1 cc syringes/27G needles, preloaded syringes • Oxygen supply/concentrators • Nasal prongs, headboxes, other O2 delivery systems • Pulse oximeters to assess blood oxygen levels with reusable cleanable neonatal probes. • Bilirubinometers (table top and transcutaneous) • Phototherapy lamps and eye shades • Exchange transfusion kits • Hot cots, overhead heaters	Lower-cost and more robust versions of: • Blood sugar testing for babies on low volume samples, heel pricks • Oxygen condensers, including portable options • Pulse oximeters and robust probes, including with alternative power options • Syringe drivers able to take a range of syringes • Bilirubin testing devices including lower cost transcutaneous devices • Haemoglobin and blood grouping, Rhesus Point of Care • Point of care for C-reactive protein/procalcitonin • Apnoea alarm • Phototherapy devices such as portable "bilibed" to provide both phototherapy treatment and heat
Neonatal intensive care	• Continuous Positive Air Pressure (CPAP) devices with standardised safety features	• Lower-cost robust CPAP equipment with standardised settings • Neonatal intensive care context specific "kits", e.g., district hospital with ongoing support for quality use and for equipment maintenance • Surfactant as more stable, lower cost preparations

Source: Born Too Soon, Chapter 5 [113]. Note this table refers to care after the baby is born so does not include other essential tools and technologies such as antenatal steroids, or critical commodities for the woman Data sources: (East Meets West; WHO et al., 2003; Lawn et al., 2006; 2009a; PMNCH, 2011) [40,127-130].

While most premature babies are born just a few weeks early and can be saved with the right people and simple care, for more extreme premature babies, additional skills, equipment and commodities are critical, ranging from bagand-mask and controlled IV fluid-giving sets, to CPAP and surfactant (Table 3). A premature baby suffering from RDS requires oxygen and safe monitoring of oxygen saturation levels with a pulse oximeter -- however, this equipment is often unavailable. Likewise, prevention of hearing impairment for premature babies being treated for infection with gentamicin requires dose titration and, ideally, laboratory monitoring of gentamicin levels, which is often unavailable. The UN Commission on Life-saving Commodities for Women and Children has prioritized high-impact, neglected commodities, and these include several for the care of premature babies [97] (Table 4).

**Table 4:** High impact, low cost interventions to save newborns.

Intervention	Lives saved	Cost
Case management of neonatal sepsis*	~500,000	$0.13 $2.03
Chlorhexidine umbilical cord cleaning*	*Cannot estimate in LiST*	$0.23
Neonatal resuscitation*	~230,000	~$0.50 - $10.00
Antenatal corticosteroids for preterm labour*	~430,000	~$0.60
Kangaroo Mother Care	~450,000	

* Prioritised by the UN Commission on Life Saving Commodities for Women and Children

Source: interventions marked with* (Save the Children, 2013) [133]; Kangaroo Mother Care analysis (Lawn et al. 2013) [2].

Addressing newborn care in district hospitals is a key priority for improving newborn survival and health. In most countries, district hospitals are understaffed and poorly resourced compared to teaching hospitals. Design and implementation of context-specific hospital newborn care packages is critical, especially as more births occur in facilities, also with referral transport and communications linkages between home and hospital. Newborn units at this level should aim at providing warmth (using KMC, or radiant warmers), assisted enteral feeding of expressed breast milk (by feeding tube, spoon, 'paladai'), intravenous fluids for sick babies, antibiotics, oxygen, and, if possible, CPAP. There are a number of large-scale examples of improved newborn care in district hospitals including a network in rural Western Kenya [104]. In Limpopo, South Africa, a network of more than 30 district hospitals instituted an accreditation scheme and targeted quality improvement with mentor teams [105], and in another province, KwaZulu-Natal, a program called Neonatal Experiential Leaning reaches 16 hospitals with standard guidelines, resuscitation workshops, a 2-week neonatal training course, and monthly mentor visits.

Across several Indian states, peripheral hospitals have developed a dedicated newborn care space ("Newborn corner") including basic equipment, while referral hospitals have upgraded special-care baby units. According to the government's most recent data, there are now 13,219 newborn care corners, 1,574 newborn stabilisation units and 448 special newborn care units. In an attempt to remove financial barrier to neonatal care, India has introduced a program (Janani Shishu Suraksha Karyakaram) that entitles all pregnant women and neonates to free care at public facilities including free drugs and free transport from and to home. An evaluation of the special newborn care units (SNCU) concluded that it is possible to set up and manage quality SNCUs and improve the survival of small and preterm newborns and those with sepsis, although several challenges relating to human resources, maintenance of equipment, and asepsis remain [106]. Skilled and motivated nurses are the key to successful neonatal units. Some also have experimented with the use of alternative cadres of ward aides specially trained in newborn care and restricted from rotations to other wards [107].

## Priority research for care of the premature newborn

Although 92% of premature babies are born in low- and middle-income countries and 99% of premature babies in these countries die, to date the vast majority of published research has been conducted in high-income countries [108]. Important health gains are achievable in the short term with delivery or implementation research, prioritising the highest-impact interventions and the most significant constraints to scale up (Table 5) [109]. For preterm birth, there is a major gap in developing, delivering, and testing community-based interventions. A recent systematic exercise ranked 55 potential research questions to address preterm birth and stillbirth at the community level and 29 experts applied a standardised scoring approach developed by the Child Health and Nutrition Research Initiative [110]. The 10 top-ranked questions were all about delivery of interventions and implementation research, notably demand approaches, such as overcoming financial barriers and use of incentives, as well as supply, such as community health workers' tasks and supervision. The need for simplified, validated methods to identify premature babies at community level was ranked second of 55. Since the exercise was focused at community level, equipment and facility-based innovations were not listed but are widely recognised to be of critical importance (Table 5). Most equipment is developed for high-income countries and requires development and testing in varying contexts in low- and middle-income countries [111]. Discovery research often requires a longer time frame but potentially could have high return, especially with prevention of preterm birth. Description research is also important, especially to address major data gaps for impairment outcomes in low- and middle-income settings and promote more controlled assessment of some interventions, notably the impact of thermal care practices on mortality and morbidity [9].

**Table 5:** Research priorities for reducing deaths and disability in preterm babies.

**Description** • Standardised, simplified metrics for assessing acute morbidities in premature babies and tools and protocols for comparable follow up of impairment and disability in premature babies **Discovery** • Biomarkers of neonatal sepsis • Sensitive, specific identification of sepsis in preterm and other newborns • Shorter course antibiotics, oral, fewer side effects • Stability of oral surfactant **Development** • Development of simpler, lower-cost, robust devices (See Table 5.3 for full list) • Simplified identification of preterm babies in communities, increased accuracy of GA in facilities • Community initiation of Kangaroo Mother Care **Delivery** Implementation research to understand and accelerate scaling up of facility based care: • KMC, including quality improvement, task shifting • Feeding support for preterm babies • Infection case management protocols and quality improvement • Improved care of RDS, including safe oxygen use protocols and practices • Infection prevention Implementation research at community level • Simplified improved identification for premature babies • Referral strategies • Feasibility and effect of home care for preterm babies in humanitarian emergencies or where referral is not possible

Source: Born Too Soon, Chapter 5 [113].

## Prescription for action

**Figure 7:**
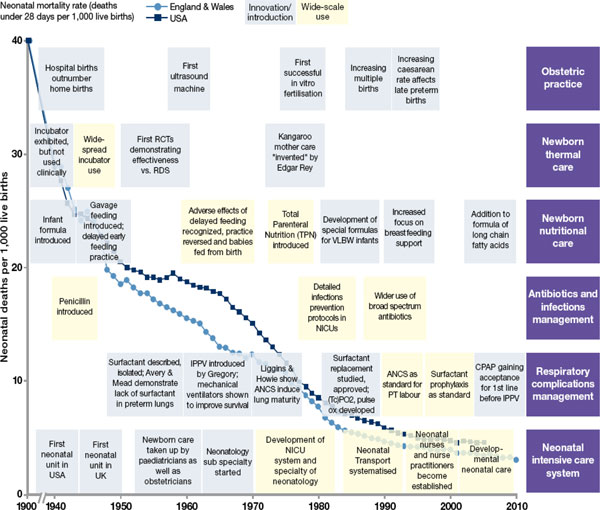
**The history of neonatal care in the United Kingdom and the United States shows that dramatic declines in neonatal mortality are possible even before neonatal intensive care is scaled up**. Source: Born Too Soon, Chapter 5 [113]. Acroynms used: ANCS = antenatal corticosteroids, CPAP = continuous positive airways pressure, NICU = neonatal intensive care, IPPV = intermittent positive pressure ventilation, VLBW = very low birth weight. Data sources: (Smith et al., 1983; NIH, 1985; Baker, 2000; Wegman, 2001; Philip, 2005; Jamison et al., 2006; Lissauer and Fanaroff, 2006; CDC, 2012; Office for National Statistics, 2012) [118-126] with thanks to Boston Consulting Group for help with the layout.

The neonatal mortality rate (NMR) in the United Kingdom and the United States was reduced to below 15 per 1,000 live births before neonatal intensive care was widely available, and the largest reduction in NMR from 40 to 15 was related to obstetric care and simpler improvements in individualised newborn care such as warmth, feeding, infection prevention, and case management (Figure 7).

Seven low- and middle-income countries have halved their preterm deaths within a decade [2]. These countries are Sri Lanka, Turkey, Belarus, Croatia, Ecuador, El Salvador, Oman and China. Some of these countries also had fertility rate reductions, which may have contributed [112], but the likely explanation is national focus on improved obstetric and neonatal care, and systematic establishment of referral systems with higher capacity of neonatal care units and staff and equipment helped in some cases by larger national budgets [2]. Over time, as neonatal care increases in scope, people skills, commodities and equipment become more critical and at a NMR below 15 per 1,000 live births, intensive care plays an increasing role. Hence low- and middle-income countries should be able to halve the risk of their newborns, their most vulnerable citizens, of dying with the right people and the right basic commodities. Yet human resource planning has not addressed this key need, and courses for nurse training in neonatal care are rare in sub-Saharan Africa and much of South Asia. Investing in frontline workers and skills is crucial to overcoming nervousness of many workers when looking after tiny babies, and building their lifesaving skills. A phased approach, for example using KMC as an entry point to show that babies under 1,000g at birth can and do survive and thrive can be a turning point for clinical staff as well as also hospital management.

Starting from existing program platforms at community level (e.g. home visit packages, women's groups) and at facility level to ensure effective care for all births at health facilities, is cost effective and more likely to show early results. However whilst families remain unreached, for example because of financial barriers to facility birth care, these gaps often mean those most at risk are unreached.

**Figure 8:**
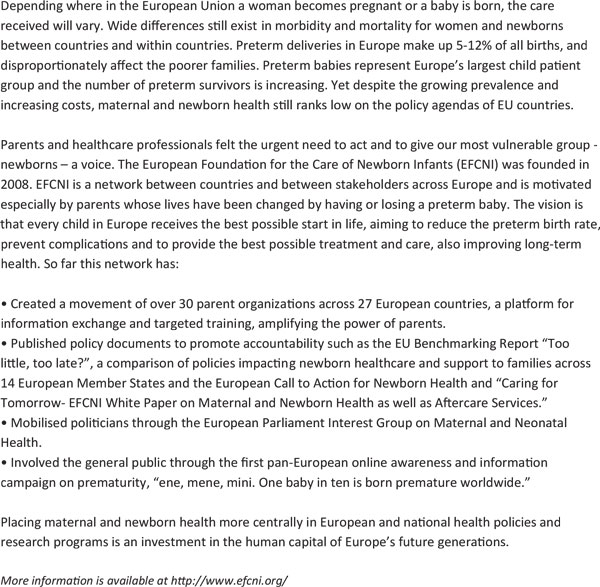
**Parents' pain and parents' power**. Source: Born Too Soon, Chapter 5.

Action for preterm birth will start from increased visibility and recognition of the size of the problem -- deaths, disability, later chronic disease, parent suffering, and wider economic loss (Table 6). In many higher-income countries, visibility is driven by empowered parents, professionals or a synergy of the two (Figure 8). Parents of premature babies are both those who experience the greatest pain and those who hold the greatest power for change. Societal mobilisation has made it unacceptable for women to die while giving birth. The voice of women and families in low-income countries is yet to be mobilised for the issue of newborn deaths and stillbirths, and these deaths too often continue to be accepted as the norm despite the existence of highly cost-effective and feasible solutions.

**Table 6:** Actions for reducing deaths and disability in preterm babies.

**Invest and plan** ***Assess and advocate for newborn and preterm baby care, mobilise parent power*** • Review existing policies and programs to integrate high-impact care for premature babies • Train nurses for newborn care and include skilled personnel for premature baby care in human resource planning for all levels of the health system where babies are cared for • Ensure essential equipment and commodities are consistently available **Implement** ***Seize opportunities through other programs including*** For all facility births ensure: • immediate essential newborn care and neonatal resuscitation if needed • infection prevention and management At community level scale up: • Pregnancy and postnatal home visits, including behaviour change messages for families, as well as identification, extra care and referral for premature babies, • Breastfeeding promotion through home visits, well baby clinics, baby friendly hospital initiative ***Reach high coverage with improved care for premature babies especially*** • Kangaroo Mother Care and improved feeding for small babies • Antenatal corticosteroid use • Respiratory distress syndrome support, safe oxygen use • Audit and quality improvement processes • Provide family support Where additional capacity consider: • Additional neonatal care such as CPAP, • Referral level neonatal intensive care, with safeguards to ensure the poor can also access this care Careful attention to follow up of premature babies (including extremely premature babies) and early identification of impairment **Inform and improve program, coverage and quality** • Improve the data including morbidity follow up and use this in programmatic improvement e.g. gestational specific survival, rates of retinopathy of prematurity etc. • Address key gaps in the coverage data especially for Kangaroo Mother Care **Innovate and undertake research** • Establish prioritised research agenda with emphasis on implementation • Invest in research and in research capacity • Conduct multi-country studies of effect, cost and "how to" and disseminate findings linked to action

Source: Born Too Soon, Chapter 5 [113].

## Conclusion

Globally, progress is being made in reducing maternal deaths and child death after the first month of life. Progress for neonatal deaths is slower. Severe neonatal infection deaths may possibly be reduced through "trickle down" from child health programs. Neonatal deaths due to intrapartum complications ("birth asphyxia") are also beginning to decline, although slowly, perhaps related to increased investments in care at birth and maternal health and care. However the over 1 million deaths among premature babies are less likely to be reduced though "trickle down" from other programs, and indeed it was the specific vulnerability and needs of the premature baby that catalyzed the specialty of neonatology. There are simple solutions that will reduce deaths among premature babies immediately for the poorest families at home in the lowest income settings -- for example promotion of early and exclusive breastfeeding, and handwashing, chlorhexidine cord applications and skin-to-skin care. Women's groups and other community mobilisation approaches are key; however, higher-impact care in facilities is also needed, such as KMC, feeding support and KMC and management of infections and respiratory complications and this is dependent on nurses and others with skills in caring for small babies, as well as more innovative technology, and can be phased over time to add increased complexity. Starting with intensive care will fail if simple hygiene, careful attention to feeding and other basic building blocks are not in place. Many countries cannot afford to rapidly scale up neonatal intensive care but no country can afford to delay doing the simple things well for every baby and investing extra attention in survival and health of newborns especially those who are preterm.

## List of abbreviations used

CPAP: Continuous positive airway pressure; KMC: Kangaroo Mother Care; PMNCH: Partnership for Maternal, Newborn and Child Health; RDS: Respiratory distress syndrome; SNCU: Special newborn care units; WHO: World Health Organization.

## Competing interests

The authors declare that they have no conflict of interest

## Authors' contribution

The chapter was drafted by JEL with MVK and all other authors reviewed and contributed.

## Funding

The time of JEL and MVK was funded by a grant from Bill & Melinda Gates Foundation to Save the Children's Saving Newborn Lives programme. The Born Too Soon report was funded by March of Dimes, the Partnership for Maternal, Newborn and Child Health and Save the Children.

## Supplementary Material

Additional file 1 **In line with the journal's open peer review policy, copies of the reviewer reports are included as additional file **1. Click here for file
