# Supplementary material for: Born Too Soon: Care for the preterm baby
Source: Reprod Health. 2013 Nov 15;10(Suppl 1):S5. doi: 10.1186/1742-4755-10-S1-S5 (PMC3828583; doi:10.1186/1742-4755-10-S1-S5)
Supplement: Additional file 1 — In line with the journal's open peer review policy, copies of the reviewer reports are included as additional file 1. [file 1742-4755-10-S1-S5-S1.doc]

**Referee’s comments to the authors– this sheet WILL be seen by the author(s) and published with the article**

| Title | **Care for the preterm baby** |
| --- | --- |
| Author(s) | Joy E Lawn, Ruth Davidge, Vinod K Paul, Severin von Xylander, Joseph de Graft Johnson, Anthony Costello, Mary Kinney, Joel Segre and Liz Molyneux |
| Referee’s name | Gerard Breart |

**When assessing the work, please consider the following points, where applicable:**

1. Is the question posed by the authors new and well defined?
2. Are the methods appropriate and well described, and are sufficient details provided to replicate the work?
3. Are the data sound and well controlled?
4. Does the manuscript adhere to the relevant standards for reporting and data deposition?
5. Are the discussion and conclusions well balanced and adequately supported by the data?
6. Do the title and abstract accurately convey what has been found?
7. Is the writing acceptable?

Please make your report as constructive and detailed as possible in your comments so that authors have the opportunity to overcome any serious deficiencies that you find and please also divide your comments into the following categories:

- Major Compulsory Revisions (which the author must respond to before a decision on publication can be reached)
- Minor Essential Revisions (such as missing labels on figures, or the wrong use of a term, which the author can be trusted to correct)
- Discretionary Revisions (which are recommendations for improvement but which the author can choose to ignore)

Where possible please supply references to substantiate your comments.

When referring to the manuscript please provide specific page and paragraph citations where appropriate.

**General comments:This is an excellent paper with numerous references ,it will be useful for those who care for preterm babies**

**Major compulsory revisions:**

**Minor essential revisions:**

**Discretionary revisions:**

**Even if the title of the paper is “care for the preterm baby”, it might be good to say more about prevention of complications , in particular by the use of antenatal corticosteroids which can almost be considered**

*(continue on the next sheet)*

Considered as care of pretrem since they are administered in case of “imminent preterm delivery.

Place of birth and care is still debated and very few is said in this paper about this which is an important issue for organisation of care.

The paper concentrates on neonatal care.Can something be said on post natal care for screening , prevention or diagnosis of handicap. This might be considered as out of the scope of the paper. In that case it should be specified

**Referee’s comments to the authors– this sheet WILL be seen by the author(s) and published with the article**

| Title | **Care for the preterm baby** |
| --- | --- |
| Author(s) | Joy E Lawn, Ruth Davidge, Vinod K Paul, Severin von Xylander, Joseph de Graft Johnson, Anthony Costello, Mary Kinney, Joel Segre and Liz Molyneux |
| Referee’s name | Ramkumar Menon |

**When assessing the work, please consider the following points, where applicable:**

1. Is the question posed by the authors new and well defined?
2. Are the methods appropriate and well described, and are sufficient details provided to replicate the work?
3. Are the data sound and well controlled?
4. Does the manuscript adhere to the relevant standards for reporting and data deposition?
5. Are the discussion and conclusions well balanced and adequately supported by the data?
6. Do the title and abstract accurately convey what has been found?
7. Is the writing acceptable?

Please make your report as constructive and detailed as possible in your comments so that authors have the opportunity to overcome any serious deficiencies that you find and please also divide your comments into the following categories:

- Major Compulsory Revisions (which the author must respond to before a decision on publication can be reached)
- Minor Essential Revisions (such as missing labels on figures, or the wrong use of a term, which the author can be trusted to correct)
- Discretionary Revisions (which are recommendations for improvement but which the author can choose to ignore)

Where possible please supply references to substantiate your comments.

When referring to the manuscript please provide specific page and paragraph citations where appropriate.

**General comments:**

Care for the preterm baba by Lawn et al

The chapter has elegantly identified global issues dealing with caring for premature babies, reports existing care in low, middle and high income countries, then proposes strategies for caring premature babies. In line with Born Too Soon report published in 2012 and the goals to be established by 2015, this chapter is in line with the second edition of Born Too Soon Report.

The key areas identified include;

- Difficulties to implement strategies in many parts of developing world
- Lack of RCTs to implement evidence based medicine
- Globalization of evidence based medicine where many trials are conducted in North America and Europe and how impractical they are to implement
- Potential implementation of minimal but essential care that are proven successful and cost effective in developing world

**Major compulsory revisions:**

**Minor essential revisions:**

**Please see my comments to the authors**

**Discretionary revisions:**

*(continue on the next sheet)*

*Continued*:

However, there are several areas that can improve

- Although a brief mentioning of pitfalls of evidence based medicine has been made in the chapter, it is important to point out the gaps in the knowledge base to implement such strategies.
- The evidence based medicine practices reported in the chapter most of which based on RCTs in USA and Western Europe and globalized by NIH ignores the difficulties in geo-political-social-cultural setup in implementing such strategies or programs in the most needed parts of the world where care is much needed. This globalization is one of the major limitations of preterm birth prevention or premature baby care programs.
- Authors classify prematurity based on gestational age as the severity is different between very early (< 28 weeks), early (28-32weeks) and late (> 32 and < 37 weeks) but the care should not be the same for babies born to ‘indicated vs. iatrogenic’ preterm births as it is unlikely that RDS or any other complications and the biomolecular and biomechanical pathways leading to underlying pathologies in each of these conditions are the same. So care for these neonates cannot be the same although the clinical presentation may be same but underlying pathways may be different. A success of 50% or less in many of the RCTs in developing world is partly attributable to the lack of understanding of true risk factors and the risk induced pathology
- Evidence based medicine can survive only if knowledge gaps are filled and chapter should identify gaps beyond the epidemiological and statistical data and call for implementation strategies that are specific for each region, country, and continent.
- Corticosteroids administration has been mentioned, this is a prime example of having a live baby with minimal impact during early stages of life. By suppressing the inflammatory pathways this is achieved but the scientific world ignores the fetal programing changes induced by steroid administration at early stages of life. RCTs are incredibly successful with glucocorticoids as they only report one major outcome, namely reduction of RDS and ignore other short and long term consequences. Accelerated ageing of the organ systems and modification of many of proteins (lysine acetylation is a well-documented phenomenon of glucocorticoids) at birth or during early stages of life may be detrimental to a premature baby during the course of life. I urge authors to include a note on much needed follow up study of the babies (not just in US and Western Europe) in low and middle income countries and how these may cause metabolic derangements in their life course.
- I suggest detailing the limitations of RCTs and calling for more systematic reviews to fill knowledge gaps. As long as they exist, the strategies implemented will provide short term success with long term consequences and more burdens to the global community to prematurity.

## Comments from the Editor:

For all the articles, these should be seen as independent papers that can be read without the need to read the other papers in the series.
